# The Potential Protective Role of Mitochondrial Haplogroup R in Ovarian Response: An Exploratory Study

**DOI:** 10.3390/ijms26062513

**Published:** 2025-03-11

**Authors:** Yun Cheng, Cheng-Rung Huang, Yin-Hua Cheng, Yung-Chiao Chang, Pei-Ling Weng, Kuo-Chung Lan

**Affiliations:** 1 Department of Obstetrics and Gynecology, Kaohsiung Chang Gung Memorial Hospital, Chang Gung University College of Medicine, Kaohsiung 833, Taiwan; iamatree404@gmail.com; 2 Center for Menopause and Reproductive Medicine Research, Kaohsiung Chang Gung Memorial Hospital, Kaohsiung 833, Taiwan; jackie31428@gmail.com (C.-R.H.); justjudykimo@gmail.com (Y.-H.C.); maurenlab15@gmail.com (Y.-C.C.); lingpay@gmail.com (P.-L.W.); 3 Department of Obstetrics and Gynecology, Jen-Ai Hospital, Taichung 412, Taiwan

**Keywords:** mitochondrial haplogroup R, ovarian response, diminished ovarian response, mitochondrial DNA copy numbers, telomere length

## Abstract

An investigation of the mtDNA haplogroup in 96 Taiwanese women with diminished ovarian response (DOR) and normal ovarian response (NOR) showed that only the haplogroup R is less likely to experience DOR than other mtDNA haplogroups. When analyzing the relationship between age and mitochondria-related markers (mtDNA copy number, ROS levels, and telomere length), it was observed that ROS levels and telomere length exhibited age-dependent changes, and the number of retrieved oocytes decreased with age. However, in the R haplogroup, these mitochondria-related markers remained stable and did not show significant changes with age. Additionally, in the R haplogroup, the number of oocytes did not decline with age, suggesting a unique protective effect associated with this haplogroup. Our study supports the notion that the mtDNA haplogroup may serve as a biomarker for infertility in Taiwanese women.

## 1. Introduction

In recent decades, delayed parenthood has emerged due to increased life expectancy, later marriage, career prioritization, widespread contraception, and shifting social norms that emphasize individual autonomy and strategic life planning [1,2]. This trend, however, coincides with a natural decline in ovarian function with advancing age, leading to a diminished ovarian oocyte reserve and compromised oocyte quality, factors critically impacting fertility and elevating miscarriage risks [3]. The success of assisted reproductive technologies (ARTs), including in vitro fertilization, is fundamentally determined by both the number and quality of oocytes available in the ovarian reserve. Thus, a pre-assessment of these factors could significantly boost ART outcomes.

The quality of oocytes, which contain an exceptionally high number of mitochondria, depends significantly on mitochondrial activity [4,5]. Alterations in mitochondrial ATP production, mitochondrial membrane potential, and mitochondrial DNA copy numbers have been shown to affect the development, maturation, and fertility of oocytes [4,6,7,8]. Research has indicated that these functions alter with oocyte aging, as evidenced by reduced ATP levels in aged bovine granulosa cells and diminished mitochondrial DNA (mtDNA) copy numbers in mature oocytes from both cows and mice [9,10].

During ATP production, mitochondria generate reactive oxygen species (ROS) as a natural byproduct of cellular respiration. Excessive ROS (superoxide, hydroxyl, and hydrogen peroxide) can trigger oxidative stress by damaging cellular proteins, lipids, and DNA, ultimately accelerating oocyte aging and deterioration [11,12,13]. Elevated intracellular concentrations of ROS have been demonstrated to compromise oocyte developmental competence, significantly impair fertilization potential, and attenuate subsequent embryonic development, thereby contributing to reduced reproductive outcomes and infertility [14]. Antioxidant enzymes, such as superoxide dismutase, peroxiredoxin 3 (Prdx3), and thioredoxin 2 (Txn2), generally mitigate ROS effects, but their expression declines with age, resulting in increased oxidative stress in the ovaries [15,16]. Similarly, telomere dynamics offer insights into reproductive aging. Telomeres, which consist of repetitive TTAGGGn sequences, are essential for protecting the termini of DNA during the replication process. A notable reduction in telomere length has been documented in women undergoing reproductive aging, and this phenomenon has similarly been demonstrated in aged mouse oocytes [17,18,19]. It has been observed that granulosa cells exhibiting shortened telomeres contribute to a higher incidence of aneuploid embryos in mice models. In human studies, telomere length (TL) in granulosa cells showed a negative correlation with the rate of aneuploidy, particularly in the younger age group (under 38 years old) [20]. The progressive shortening of telomeres in aged oocytes, driven by mechanisms such as ROS exposure and environmental insults rather than cell cycling, leads to dysfunctional telomere maintenance and replicative senescence. This process contributes significantly to the marked decline in fertility associated with advancing age [21,22].

Genetic variations, particularly mtDNA polymorphisms, are not only associated with aging processes [23,24] but are also implicated in a diverse spectrum of pathological conditions, including autism spectrum disorder, type 2 diabetes, Parkinson’s disease, neurodegenerative diseases, osteoarthritis, endometriosis, aging, and polycystic ovary syndrome (PCOS) [25,26,27,28,29,30,31,32,33]. This is attributed to the fact that mtDNA, unlike nuclear DNA (nDNA), is not protected by histone proteins, making mtDNA more susceptible to damage by ROS, leading to the rapid accumulation of mutations in mtDNA [34]. The susceptibility of mtDNA to mutations induced by reactive oxygen species (ROS), due to its absence of protective histones, has garnered interest regarding its involvement in various pathologies and its utility in tracing genetic lineages, known as haplogroups. Haplogroups represent clusters of related mtDNA variants shared by individuals with common ancestry [31,35,36,37].

When exploring mtDNA haplogroups based on geographic regions, over 90% of European individuals and USA Caucasians were classified into 10 mtDNA haplogroups (H, I, J, K, M, T, U, V, W, and X) [38,39,40,41,42]. African populations were predominantly categorized into macrohaplogroup L, further divided into L0, L1, L2, L3, L4, L5, and L6 [39,41,43,44,45,46,47,48], while Asian populations primarily belonged to haplogroups M and N, with East Asians traced back to ten specific haplogroups (A, B, C, D, F, G, M, N, R, and Z) [49]. Beyond geographic distribution, certain haplogroups correlate with disease predispositions. For instance, the JT macrohaplogroup shows reduced prevalence in women with a diminished ovarian response (DOR) [50], whereas haplogroup H is associated with a higher risk of endometriosis [33]. Moreover, specific polymorphisms like G207A, 16036GGins, and 16049Gins in the mtDNA D-loop are linked to reduced polycystic ovary syndrome risk [32]. Based on these documented associations, this study investigates mtDNA haplogroups as prospective biomarkers for female infertility in a Taiwanese population, with particular emphasis on ovarian response. This research seeks to elucidate the influence of genetic ancestry on reproductive outcomes, potentially offering new insights into personalized fertility assessment and treatment approaches.

## 2. Results

### 2.1. Distribution of mtDNA Haplogroups and Their Association with Ovarian Response

To elucidate the relationship between mtDNA haplogroups and ovarian response, we conducted a study involving 96 female participants in Taiwan undergoing oocyte retrieval, classifying their mtDNA haplogroups. The median age of the participants was 37 years (range 21–45). The data showed the mtDNA haplogroups to be as follows: A group, 7.3%; B group, 9.4%; C group, 2.1%; D group, 17.7%; E group, 4.2%; F group, 15.7%; G group, 4.2%; M group, 24%; N group, 6.3%; R group, 8.3%; and Z group, 2.1% (Table 1). The median ages for each haplogroup are presented in Table 1. Notably, the occurrence of the R haplogroup was 0% in the DOR group compared to 10.8% in the NOR group (Table 1). This was the only haplogroup to show a significant difference between the two groups.

### 2.2. Correlations of Oocyte Quantity, Age, and Mitochondrial Biomarkers in 96 Patients and the NOR and DOR Groups

**Figure 1:**
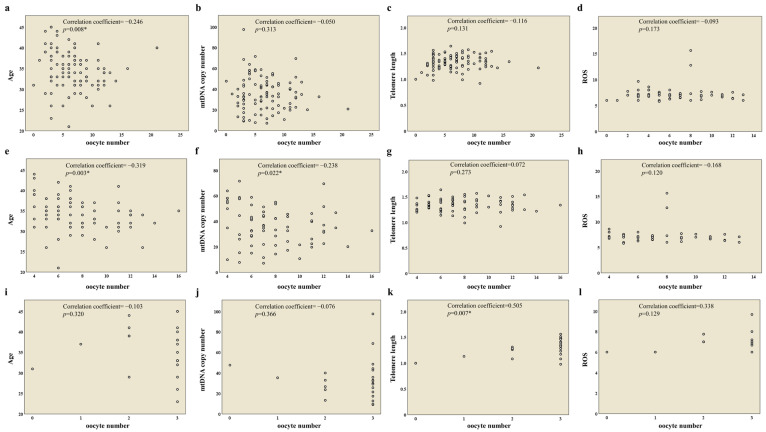
Relationships between oocyte number and age, mtDNA copy number, telomere length, and ROS levels in 96 patients (**a**–**d**), the normal ovarian response (NOR) group (**e**–**h**), and the diminished ovarian response (DOR) group (**i**–**l**). Data were analyzed via unpaired two-tailed correlation analysis (*p* < 0.05 *).

We conducted a comprehensive analysis of the correlations between oocyte quantity, age, and mitochondrial biomarkers (mtDNA copy number, telomere length, and ROS) among 96 patients and the NOR and DOR groups to explore potential mechanistic interconnections in reproductive cellular dynamics. It was found that the number of oocytes was negatively correlated with age among 96 patients (Figure 1a), while no significant association was found between the number of oocytes and other mitochondrial biomarkers (mtDNA copy number, telomere length, and ROS levels) (Figure 1b–d).

Moreover, the number of oocytes was negatively correlated with age and mtDNA copy number in the NOR group (Figure 1e,f), while a positive correlation was found between the number of oocytes and telomere length in the DOR group (Figure 1k).

**Figure 2:**
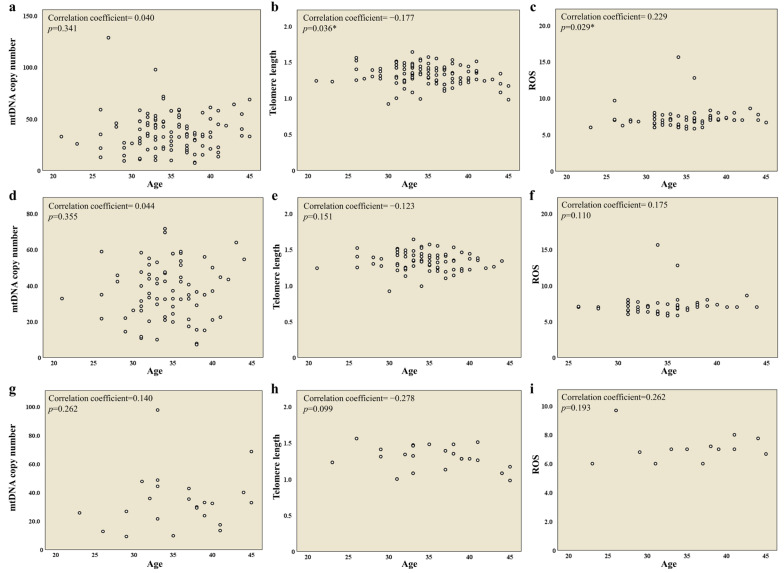
Relationships between age and mtDNA copy number, telomere length, and ROS levels were analyzed in 96 patients (**a**–**c**), the NOR group (**d**–**f**), and the DOR group (**g**–**i**). Data were analyzed via unpaired two-tailed correlation (*p* < 0.05 *).

Next, we examined the correlation between age and mitochondrial biomarkers (mtDNA copy number, telomere length, and ROS) among 96 patients and the NOR and DOR groups. The results showed a decrease in telomere length (Figure 2b) and an increase in ROS levels (Figure 2c) with age among 96 patients, while no correlation with age was observed in the NOR and DOR groups (Figure 2d–i).

### 2.3. Correlations of Oocyte Quantity, Age, and Mitochondrial Biomarkers in Haplogroups D, F, M, and R

**Figure 3:**
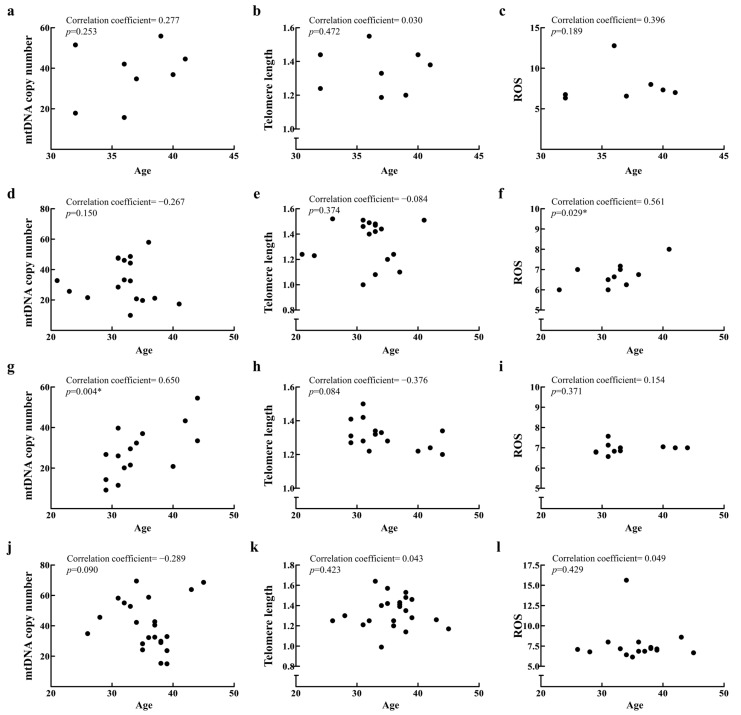
Relationships between age, mtDNA copy number, telomere length, and ROS levels were analyzed in patients from the R (**a**–**c**), D (**d**–**f**), F (**g**–**i**), and M groups (**j**–**l**). Data were analyzed via unpaired two-tailed correlation analysis. * represents a *p*-value < 0.05.

**Figure 4:**
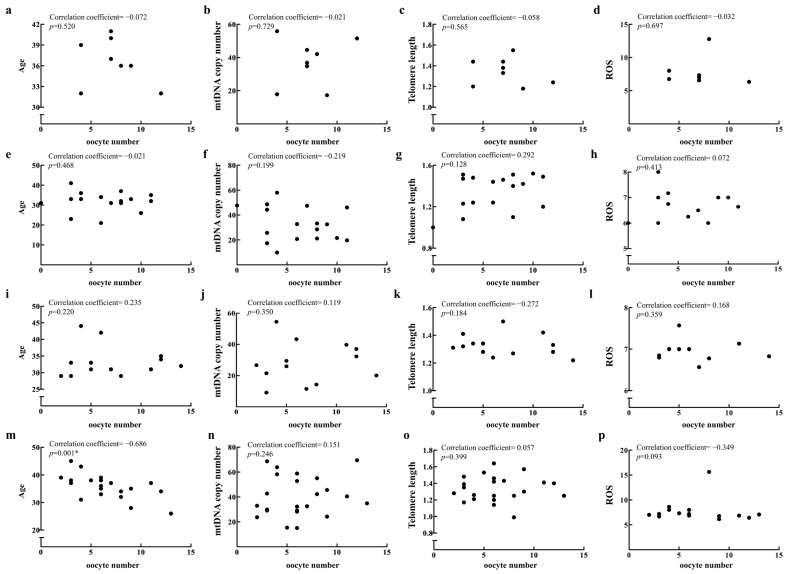
Relationships between oocyte number and age, mtDNA copy number, telomere length, and ROS levels were analyzed in patients from the R (**a**–**d**), D (**e**–**h**), F (**i**–**l**), and M groups (**m**–**p**). Data were analyzed via unpaired two-tailed correlation analysis.* represents a *p*-value < 0.05.

In addition to the R group, which showed significant findings, we also selected the D, F, and M groups for analysis due to their larger sample sizes. No significant age-related changes in mtDNA copy number, telomere length, or ROS levels were observed in the R (Figure 3a–c) and M groups (Figure 3j–l). The D group showed only an increase in ROS levels with age (Figure 3f), and the F group showed only an increase in mtDNA copy number with age (Figure 3g). Moreover, in the D, F, and R groups, oocyte number did not correlate with age (Figure 4a,e,i), and other mitochondrial biomarkers similarly showed no correlation (Figure 4b–d,f–h,j–l). Notably, oocyte number decreased with age only in the M group (Figure 4m), while other mitochondrial biomarkers showed no correlation (Figure 4n–p).

## 3. Discussion

Recent research has identified mitochondria as critical regulators in ovarian aging, modulating key hallmarks and molecular pathways through mechanisms such as mtDNA copy number, ROS levels, and telomere length [10,14,15,16,17,18,19,20,51,52,53].

mtDNA copy number, which quantifies cellular mtDNA content, correlates with mitochondrial enzyme activity and ATP production despite not directly measuring function [54]. In our investigative study, we did not observe a statistically significant correlation between mitochondrial DNA (mtDNA) copy number and chronological age. Consistent with our findings, a contemporaneous investigation focusing on Taiwanese female participants similarly reported a lack of substantial age-related variation in mtDNA copy number [20]. However, conflicting evidence exists in the literature. Animal studies have demonstrated an inverse relationship between mtDNA copy number and the aging process [10,55]. In human subjects, emerging research suggests an opposing perspective. Several studies suggest that women of advanced reproductive age and those experiencing a DOR exhibit reduced mtDNA levels compared to younger women and those with a normal ovarian reserve [56,57,58]. These divergent findings underscore the intricate nature of mtDNA dynamics.

In our study, utilizing granulosa cells as biological samples, we identified an age-dependent pattern characterized by progressive telomere shortening and significant ROS level elevation. This observation aligns with the broadly accepted notion that telomere attrition serves as a hallmark of cellular senescence and biological aging [59,60]. However, subsequent studies have reported divergent findings. Yu et al. found no significant age-related differences in the telomere lengths of both leukocytes and granulosa cells between young and advanced-age groups, noting that granulosa cell telomere length served as the sole significant biomarker for aneuploidy rates in young-age subjects [20]. Hanson et al. reported that shorter white blood cells relative to telomere length significantly correlated with increased patient age and embryonic aneuploidy rates, while they found no significant associations between cumulus cell telomere length and their analyzed variables [61]. These disparate findings may be attributed to varying study inclusion criteria and other telomere length-modifying factors beyond age, including specimen source, ethnicity, and lifestyle factors such as smoking [62,63,64]. Beyond infertility populations, studies of premature ovarian insufficiency (POI) patients demonstrate that telomere shortening in both leukocytes and granulosa cells, alongside reduced telomerase activity, is strongly associated with biochemical POI [65].

Notably, our analysis revealed no statistically significant correlation between oocyte quantity and the other mitochondrial biomarkers examined.

Evidence increasingly demonstrates that ovarian aging involves concurrent qualitative and quantitative alterations in oocyte mitochondria [55,66]. Distinct mitochondrial DNA haplogroups, defined by specific single-nucleotide polymorphisms across human populations [67], influence mitochondrial performance by modulating bioenergetics, coupling efficiency, and ROS production [28]. In our analysis, haplogroup R showed a significantly lower frequency among patients with a DOR, demonstrating complete absence (0%) compared to its 10.8% prevalence in controls (*p* = 0.007). A prior study investigated the association between mitochondrial DNA haplogroups and ovarian function within European populations. May-Panloup et al. found that, among 200 Caucasian women, the JT macrohaplogroup was significantly less frequent in patients exhibiting ovarian aging (7.4%) than in controls (20.7%, *p* = 0.006) and the general French population (18.8%, *p* = 0.0012). Furthermore, carriers of the JT macrohaplogroup exhibited enhanced ovarian reserve parameters compared to individuals with other macrohaplogroup classifications [50].

Mitochondrial haplogroup R, a sub-haplogroup of haplogroup N, is shared across populations in Europe and East Asia [68]. Previous studies have demonstrated that haplogroup R exerts protective effects against obesity [69] and serves as an independent predictor of enhanced neurological recovery in septic encephalopathy [70]. While mitochondrial DNA haplogroups are known to exhibit distinct distribution patterns across ethnic populations [38,39,41,49], this study is the first to explore the relationship between haplogroup classification and ovarian response in Asian populations.

Understanding the age-related decline in ovarian response remains a critical focus in reproductive medicine. Mitochondrial genetics has emerged as a promising avenue for investigating reproductive aging mechanisms, given the crucial role of mitochondria in fertility. Notably, our research indicates that women carrying the R haplogroup may exhibit a better preservation of ovarian response over time than carriers of other mitochondrial haplogroups. This differential impact of mitochondrial DNA variation on reproductive aging suggests that haplogroup classification could influence the trajectory of ovarian response dynamics.

The level of reactive oxygen species (ROS) in follicular fluid is a crucial microenvironmental factor for oocyte development [71]. Analyses of the ROS levels in follicular fluid can be used to assess a patient’s fertility potential. However, this assessment method also faces limitations. The timing of sampling is critical, as ROS levels in follicular fluid may vary at different stages of follicular development. Consequently, the results of ROS detection often require further investigation to fully understand their potential in improving fertility outcomes.

## 4. Materials and Methods

### 4.1. Patient Selection

This study was approved by the Institutional Review Board (IRB) of Chang Gung Memorial Hospital (protocol number: CGMH98-4000B) on 22 February 2010, and it was conducted in accordance with the Declaration of Helsinki. Participants were prospectively enrolled between 1 December 2010, and 1 December 2012. The study population comprised women receiving assisted reproductive treatments, specifically in vitro fertilization (IVF) or intracytoplasmic sperm injection (ICSI), at the Department of Obstetrics and Gynecology, Chang-Gung Memorial Hospital. All enrolled couples underwent comprehensive infertility evaluation protocols. Patients were not selected for age, sperm parameters, infertility criteria, or individual characteristics such as BMI, smoking status, or hormonal profile. The inclusion criteria were the presence of both ovaries, no history of ovarian surgery, and no ovarian abnormalities as assessed by a transvaginal ultrasound. The exclusion criteria were the presence of ovarian cysts and inaccessible ovaries and cycles for oocyte donation. All study participants were notified about the utilization of granulosa cells (GCs) and follicular fluid for the research. All participants provided written informed consent. A diminished ovarian response (DOR), which demonstrates poor reproductive outcomes even with assisted reproductive technology (ART), was defined in this study by a threshold of ≤3 oocytes retrieved per treatment cycle [72,73].

### 4.2. Granulosa Cell (GC) Collection and Follicular Fluid (FF)

The laboratory facilities, clinical strategy, and protocol for controlled ovarian hyperstimulation followed the standard downregulation regimen that we previously published. All patients followed a stimulation protocol using the gonadotropin-releasing hormone (GnRH) agonist method for controlling ovarian stimulation [74,75]. Recombinant follicle-stimulating hormone (rFSH) and/or recombinant luteinizing hormone (LH) or urinary follicle-stimulating hormone (uFSH) were administered continuously until at least two follicles reached a diameter of 18 mm or greater, accompanied by adequate serum estradiol (E2) levels. Upon achieving optimal follicular maturation, human chorionic gonadotropin (hCG) (Ovidrel^®^; Serono, Modugno, Italy) was administered for the final maturation trigger. Oocyte retrieval and concomitant granulosa cell collection were subsequently performed 34–36 h following hCG administration.

### 4.3. Preparation of Granulosa Cells (GCs) and Total DNA Extraction

After oocyte retrieval, granulosa cells were collected and pooled from each individual woman. The pooled granulosa cells were then transferred to a separate dish containing phosphate-buffered saline (PBS). A portion of the pooled granulosa cells was centrifuged to separate them from blood cells or other contaminants. These cells were washed three times before DNA extraction. Total DNA extraction from the granulosa cells was performed using a DNeasy Blood and Tissue Kit (Qiagen, Valencia, CA, USA), adhering to the manufacturer’s protocol. In summary, the granulosa cells were lysed using a lysis buffer, and the resulting sample was then applied to NucleoSpin tissue columns. Following the addition of chaotropic salts and ethanol, DNA adhered to the silica membrane. Various buffers were used to wash the sample in order to prevent contamination. The DNA was then eluted with 100 μL of elution buffer, and the concentration of the extracted DNA was assessed using a NanoDrop (Thermo Scientific, Wilmington, MA, USA).

### 4.4. Reactive Oxygen Species (ROS) Levels in Individual Follicular Fluids

Reactive oxygen species (ROS) levels were quantified in freshly aspirated follicular fluid utilizing a chemiluminescence assay, with luminol (5-amino-2,3-dihydro-1,4-phthalazinedione) serving as the detection probe. For this, 400 μL of clear supernatant was dispensed into each well of a 96-well microplate. Next, 10 μL of luminol solution (5 mM in DMSO) was dispensed into each well. Each sample was analyzed over a duration of 10 min. The ROS values were quantified and are presented as counted photons per second (cps).

### 4.5. Quantification and Analysis of mtDNA Copy Number and Telomere Length

To explore the potential mechanisms involved in mitochondrial bioenergetics and biogenesis during ovarian aging, markers such as mtDNA copy number and telomere length serve as indicators of cell aging and oocyte quality. We determined the mtDNA copy number in the granulosa cells by using a quantitative polymerase chain reaction (qPCR) protocol [20]. Amplifications were performed using Fast SYBR Green Master Mix (Applied Biosystems, Vilnius, Lithuania), and reactions were conducted in an ABI 7500 Fast Real-Time PCR System (Applied Biosystems, Vilnius, Lithuania). Specific primer sequences were used to determine the mtDNA copy number: the forward primer was 5′-CCTCTAGAGCCCACTGTAAAGCTAAC-3′, and the reverse primer was 5′-TTTAGTTGGGTGATGAGGAATAGTGTA-3′. The PCR cycles for the mtDNA copy number reactions involved initial denaturation at 95 °C for 10 min, followed by 40 cycles of 94 °C for 15 s and 60 °C for 1 min. The raw fluorescence data were analyzed to obtain threshold cycle (Ct) values for triple repeats of each sample. The 2^−ΔΔCT^ method was employed to compare the Ct values with an endogenous reference gene, β-actin, for mtDNA quantification via qPCR. ΔCt values were calculated by subtracting the average β-actin Ct value from the average mtDNA Ct value (ΔCt = mtDNA Ct − β-actin Ct). We determined the mtDNA telomere length in granulosa cells using a modified quantitative qPCR protocol, as outlined in previous studies [76]. For a telomere length analysis, a 25 μL PCR reaction mixture was prepared with 100 ng of each DNA extract. Different primer sequences were employed: the forward primer was 5′-GGTTTTTGAGGGTGAGGGTGAGGGTGAGGGTGAGGGT-3′, and the reverse primer was 5′-TCCCGACTATCCCTATCCCTATCCCTATCCCTATCCCTA-3′. The telomere PCR protocol consisted of initial denaturation at 94 °C for 1 min, followed by 30 cycles of 95 °C for 15 s and 56 °C for 1 min. The single-copy gene 36B4 was used as a universal reference for gene expression and telomere measurement. The primer sequences for 36B4 were as follows: the forward primer was 5′-CAGCAAGTGGGAAGGTGTAATCC-3′, and the reverse primer was 5′-CCCATTCTATCATCAACGGGTACAA-3′. The PCR cycles for the 36B4 gene included initial denaturation at 94°C for 1 min, followed by 30 cycles of 95 °C for 15 s, 56 °C for 20 s, and 72 °C for 20 s. Telomere length was measured as the ratio between the telomere and single-copy gene (T/S ratio).

### 4.6. Annotation and Selection of Various Polymorphisms for Haplogroup Classification of Granulosa Cells

To investigate the potential role of specific mtDNA haplogroups in ovarian aging, we examined the distribution of different haplogroups among women. DNA was extracted using a DNeasy Blood and Tissue Kit (Qiagen, Valencia, CA, USA), following the manufacturer’s protocols. The DNA samples were then sent to the Genomic & Proteomic Core Laboratory at Kaohsiung Chang Gung Memorial Hospital for further processing, and a next-generation sequencing (NGS) library was prepared using a Mitochondrial LibPrep/TE Kit (Twist, South San Francisco, CA, USA), following the manufacturer’s protocol. The constructed library was then analyzed by the Genomic Medicine Core Laboratory at Linkou Chang Gung Memorial Hospital. Additionally, mtDNA variants were annotated for the identification of mtDNA haplotypes using Haplogrep 3 (https://haplogrep.i-med.ac.at (accessed on 18 February 2025).).

### 4.7. Statistical Analysis

All statistical analyses were performed using SPSS software (version 25), while data visualization was conducted using GraphPad Prism software (version 5.0). Categorical variables are summarized using proportions. To control for confounding factors, we conducted a multivariate logistic regression analysis, using oocyte number as the dependent variable and the mtDNA haplogroups as the independent variables. The findings are presented as odds ratios (ORs) with 95% confidence intervals (CIs).

Additionally, a Spearman correlation analysis was conducted to examine the relationships between age, mtDNA copy number, telomere length, and reactive oxygen species (ROS) levels. Statistical significance was set to *p* < 0.05 for all analyses.

## 5. Conclusions

Our study found that mtDNA haplogroup R is more prominent in individuals with a normal ovarian response, indicating that it is a strong independent predictor against a DOR. Additionally, we observed no significant correlation between mtDNA copy number and age, while age-related patterns such as telomere shortening and elevated ROS levels were evident in granulosa cells, consistent with cellular aging processes. These findings emphasize the complex role of mitochondrial genetics in ovarian aging and offer promising insights for personalized approaches in reproductive medicine.

## 6. Limitations

Several limitations of our study warrant consideration. First, our findings are based on a relatively small sample size, necessitating validation through larger, independent cohort studies. Second, our definition of a DOR relied solely on oocyte yield as the primary outcome measure, without incorporating other established ovarian reserve markers such as anti-Müllerian hormone (AMH) levels or follicle-stimulating hormone (FSH) measurements. Future investigations incorporating these additional biochemical parameters would provide a more comprehensive assessment of the relationship between mitochondrial haplogroups and the ovarian reserve status.

Additionally, this study included couples who underwent a standardized infertility evaluation without specific selection criteria related to maternal age, semen quality, or the cause of infertility, which may have introduced variability in the results. Furthermore, in another unpublished study, we also found an association between ovarian reserve and organophosphate flame retardants in women of childbearing age. However, this study did not examine potential environmental factors that could contribute to a DOR, limiting our understanding of their impact on haplogroup and ovarian response. Lastly, the measurement of ROS levels in follicular fluid, while informative, is limited by its single time-point assessment, as ROS levels may vary during follicular development. Further studies are needed to explore the dynamics of ROS levels over time and their implications for fertility outcomes.

## Figures and Tables

**Table 1:** Multivariate logistic regression analysis of mitochondrial haplogroups associated with ovarian response in 96 patients. Age: median (range).

Haplogroup	Total	Age (years)	NOR	DOR	Multivariate	
	N = 96		N = 74	N = 22	Odds Ratio (95%CI)	*p*
A	7 (7.3%)	35.0 (31–45)	5 (6.8%)	2 (9.1%)	1.372 (0.48–3.94)	0.7824
B	9 (9.4%)	33.0 (26–44)	6 (8.2%)	3 (13.6%)	1.762 (0.69–4.48)	0.2367
C	2 (2.1%)	29.0 (28–30)	2 (2.7%)	0 (0%)	0.000 (0.01–3.93)	0.2461
D	17 (17.7%)	33.0 (21–41)	12 (16.4%)	5 (22.7%)	1.519 (0.74–3.13)	0.3548
E	4 (4.2%)	32.5 (29–35)	4 (5.4%)	0 (0%)	0.000 (0.00–1.69)	0.0594
F	15 (15.7%)	32.5 (29–44)	11 (14.9%)	4 (18.2%)	1.271 (0.59–2.73)	0.6951
G	4 (4.2%)	37.0 (34–40)	4 (5.4%)	0 (0%)	0.000 (0.00–1.69)	0.0594
M	23 (24%)	37.0 (26–45)	17(23%)	6 (27.3%)	1.257 (0.65–2.42)	0.784
N	6 (6.3%)	36.5 (33–40)	4 (5.4%)	2 (9.1%)	1.754 (0.57–5.41)	0.5672
R	8 (8.3%)	37.0 (32–41)	8 (10.8%)	0 (0%)	0.000 (0.00–0.75)	0.0007 *
Z	1(2.1%)	38.0	1 (1.4%)	0 (0%)	0.000 (0.01–9.55)	0.4974

* represents a *p*-value < 0.05.

## Data Availability

The data generated/analyzed in this study are available from the corresponding author upon request.
